# A Girl with Autoimmune Cytopenias, Nonmalignant Lymphadenopathy, and Recurrent Infections

**DOI:** 10.1155/2012/196417

**Published:** 2012-11-21

**Authors:** Marjolein A. C. Mattheij, Ellen J. H. Schatorjé, Eugenie F. A. Gemen, Lisette van de Corput, Peet T. G. A. Nooijen, Mirjam van der Burg, Esther de Vries

**Affiliations:** ^1^Department of Pediatrics, Jeroen Bosch Hospital, P.O. Box 90153, 5200 ME 's-Hertogenbosch, The Netherlands; ^2^University Medical Centre Antwerp, Antwerp, Belgium; ^3^Radboud University Medical Centre Nijmegen, Nijmegen, The Netherlands; ^4^Laboratory for Clinical Chemistry and Hematology, Jeroen Bosch Hospital, P.O. Box 90153, 5200 ME 's-Hertogenbosch, The Netherlands; ^5^Department of Medical Immunology, University Medical Center Utrecht, P.O. Box 85500, Utrecht, The Netherlands; ^6^Department of Pathology, Jeroen Bosch Hospital, P.O. Box 90153, 5200 ME 's-Hertogenbosch, The Netherlands; ^7^Department of Immunology, Erasmus Medical Center, P.O. Box 2040, 3000 CA Rotterdam, The Netherlands

## Abstract

We describe a girl, now 9 years of age, with chronic idiopathic thrombocytopenic purpura, persistent nonmalignant lymphadenopathy, splenomegaly, recurrent infections, and autoimmune hemolytic anemia. Her symptoms partly fit the definitions of both autoimmune lymphoproliferative syndrome (ALPS) and common variable immunodeficiency disorders (CVIDs). Genetic analysis showed no abnormalities in the ALPS-genes *FAS, FASLG*, and *CASP10*. The CVID-associated *TACI* gene showed a homozygous polymorphism (Pro251Leu), which is found also in healthy controls.

## 1. Introduction

Acute idiopathic thrombocytopenic purpura (ITP) is a well-known clinical entity in children. Generally, in children the disease is self-limiting and easily distinguished from a hematological malignancy, even without investigating the bone marrow [1]. However, the case becomes more complicated when the ITP becomes chronic and accompanying profound lymphadenopathy develops. We describe the diagnostic dilemma in a girl with these problems, who with time also developed recurrent respiratory infections, suffered from a prolonged episode of intractable diarrhea, a severe episode of varicella zoster infection and autoimmune hemolytic anemia.

## 2. Patient

The girl, now 9 years of age, is the second child of healthy nonconsanguineous Caucasian parents. She was born after an uncomplicated pregnancy and delivery and showed normal growth and development. Her family history reveals allergy on the paternal side and autoimmune disease and malignancies on the maternal side. At the age of 14 months, she developed ITP and showed a partial slow recovery after 3 days of high-dose intravenous immunoglobulins (IVIGs) followed by prednisolone. One year later, she suffered a relapse during a mild parainfluenza type 3 infection and treatment with 3 days of high-dose IVIG was started again. A few days after receiving this second course of high-dose IVIG she developed cervical, axillary and inguinal lymphadenopathy and enlarged tonsils: this lymphadenopathy never resolved. There was no hepatomegaly, splenomegaly, or mediastinal or abdominal lymph node enlargement at that time. Blood tests showed a mild normocytic anemia (Hb 6.4 mmol/L, MCV 77 fL) and granulocytopenia (0.7–1.0 × 10^9^/L) and large unstained cells in the hematology analyzer (8%; 0.4 × 10^9^/L). A bone marrow aspirate and biopsy showed some atypical lymphocytes and specific maturational disturbances, but no malignancy. Bone marrow immunophenotyping was normal. FAS-mediated apoptosis of T-lymphoblasts was normal (two separate tests in two different laboratories). She suffered from recurrent upper respiratory tract infections and two pneumonias in the following years and a prolonged episode of intractable diarrhea. The infection frequency improved on cotrimoxazole prophylaxis. The enlarged tonsils, cervical, axillary and inguinal lymphadenopathy and variable amounts of atypical lymphocytes in her differential remained, splenomegaly developed as well. The parents increasingly felt that she got tired more easily than other children of her age. At the age of 5 years, an adenoidectomy was performed; this procedure was combined with an excision of an inguinal lymph node for histological examination. At the age of 7 years, an episode of severe varicella zoster infection occurred which was treated successfully with 1 week of intravenous aciclovir. Five months later, she acutely developed autoimmune hemolytic anemia, which initially responded well to another course of high-dose IVIG and prednisone, but she relapsed when the prednisone was slowly tapered and stopped. Unexpectedly, her chronic thrombocytopenia improved upon this treatment. At the age of 8 years, she developed pulmonary problems with dyspnea after an (probably viral) airway infection; high resolution CT scan showed a granulomatous lymphocytic interstitial lung disease (GLILD), a form of pulmonary lymphoproliferative disease. Therapy with mycophenolic acid was started, after which she showed a slow but nearly complete pulmonary recovery; she is still slightly dyspneic upon exertion. She also developed a uveitis, which was treated with prednisolone eye drops. Now, at the age of 9 years, she is relatively stable on mycophenolic acid; stem cell transplantation is being considered.

## 3. Material and Methods

Upon her first presentation at the pediatric immunology clinic in 's-Hertogenbosch at 5 years of age, extensive investigations were performed (Table 1). Four-color immunophenotyping was performed as previously described [2, 3]. One representative paraffin-embedded tissue block of both lymph node (18 mm) and adenoid (20 mm) was selected and immunohistochemical stainings were performed (Table 2). Routine protocols for Benchmark XT Ventana were used. Kappa, lambda, and EBV were assessed using routine in situ hybridisation technique (Kappa, ISH, Ventana; lambda, ISH, Ventana; EBER). Genomic DNA was isolated from peripheral blood granulocytes using the autopure kit (Qiagen, Venlo, the Netherlands). Exon-specific M13-tagged primers were used for amplification by PCR of all coding exons including flanking regions from the genes *FAS* (NCBI NM_00043), *FASLG* (NCBI NM_000639), *CASP10* (NCBI NM_032974) and *TACI* (TNFRSF13B, NCBI NM_012452) followed by fluorescent sequencing (Applied Biosystems BigDye Terminator v1.1 Applied Biosystems). 

## 4. Results

Table 1 shows the lymphocyte subpopulations in comparison with age-matched reference values from our laboratory [2]. T-lymphocytes were slightly low to just normal, naive helper-T-lymphocytes and recent thymic emigrants were clearly decreased; double-negative TCR*αβ* ^+^ T-lymphocytes (cells without expression of CD4 or CD8 coreceptors) were normal to just increased in subsequent experiments, performed during episodes without immunosuppressive treatment.

Histological examination of lymphoid tissue revealed retention of the architectural features with a combined mild follicular hyperplasia and mild paracortical T zone expansion. In one lymph node a single granuloma was observed without necrosis or eosinophilia. Ziehl-Neelsen and other stainings for microorganisms were negative. The CD20 and CD79 positive B-cells were restricted to the normal B-cell areas. The follicular centres were highlighted by CD21 and CD23 B-cells, which stained the follicular dendritic cell meshwork. T-lymphocytes (CD2^+^, CD3^+^) showed mild expansion in the paracortical T zone. BCL2 was negative and the proliferation in MIB1 was mainly restricted to the centrocytes and centroblasts in the reactive follicular centres. Kappa and lambda revealed a polyclonal plasma cell population. ALK staining was negative, there was no pathological CD30 staining. There were no signs of Rosai Dorfman; EBV was negative. Thus, histological examination showed abnormalities, but did not point to a specific diagnosis.

Analysis of the *FAS*, *FASLG*, and *CASP10* genes revealed no abnormalities. Direct fluorescent sequencing of the *TACI* gene showed two polymorphisms, a homozygote polymorphism (p.Pro251Leu), and a heterozygote silent polymorphism.

## 5. Discussion

The girl we describe poses a diagnostic dilemma: there are several clinical entities that can be considered. Firstly, she undoubtedly fits the diagnosis of “Evans syndrome”—which is defined by the presence of at least two autoimmune cytopenias—since she suffers from chronic idiopathic thrombocytopenic purpura and autoimmune hemolytic anemia [4]. However, this is only a descriptive diagnosis that does not encompass all her features. “Evans syndrome” is increasingly being associated with specific diseases, such as autoimmune lymphoproliferative syndrome (ALPS) and common variable immunodeficiency (CVID) and, mainly in adults, lymphoproliferative disorders [4–8]. 

Since she also suffers from persistent nonmalignant lymphadenopathy and splenomegaly, ALPS is an option for this girl. ALPS is a disorder of lymphocyte apoptosis leading to chronic nonmalignant lymphoproliferation. Affected individuals often suffer from autoimmune cytopenia, splenomegaly and hepatomegaly [9]. Laboratory findings include hypergammaglobulinemia and expansion of a unique population of circulating T-lymphocytes, referred to as TCR*αβ* ^+^ double negative T-cells which owe their name to the fact that they do not express CD4 or CD8 coreceptors [10]. These T-cells respond poorly to antigens. The genetic deficit in most patients is a mutation in the *FAS* gene which encodes a cell surface receptor which, upon stimulation, induces programmed cell death [11]. The diagnostic criteria for ALPS have recently been revised. The two required criteria for the diagnosis of ALPS are (1) chronic (>6 months), nonmalignant, noninfectious, lymphadenopathy, or/and splenomegaly, (2) elevated CD3^+^TCR*αβ* ^+^CD4^−^CD8^−^ double-negative T-cells [10]. There are also four secondary accessory criteria; for a *definite* ALPS diagnosis a patient has to meet both required criteria and one of the primary accessory criteria. The diagnosis of ALPS is *probable* when two required criteria and any one of the secondary accessory criteria is present [10]. According to these criteria, our patient does not have ALPS, since she does not have consistently elevated CD3^+^TCR*αβ* ^+^CD4^−^CD8^−^ double-negative T-cells. There are several well-defined ALPS-related disorders. Of these, RALD (RAS-associated autoimmune lymphoprolipherative disease) could be an option, but our girl shows no characteristic features of this disorder such as elevations in cells of myeloid origin [10].

The girl's recurrent infections could point to possible CVID. This disease is characterized by recurrent infections and hypogammaglobulinemia. Additional clinical manifestations vary, but can include autoimmunity, splenomegaly and nonmalignant lymphoproliferation [9]. CVID is a heterogeneous group of disorders; the age of onset can be in childhood, adolescence, or even adult life. Most patients have no molecular diagnosis as yet; cases can be sporadic, or familial. There is a high incidence of hematological malignancies in CVID [12]. For a definite diagnosis, all of the following criteria need to be present: (1) onset of immunodeficiency at greater than 2–4 years of age, (2) absent isohemagglutinins and/or poor response to vaccines and (3) exclusion of defined causes of hypogammaglobulinemia. Criteria for the diagnosis of *probable* CVID are a marked decrease of IgG (at least 2 SD below the mean for age) and a marked decrease in at least one of the isotypes IgM or IgA and for *possible* CVID a marked decrease (at least 2 SD below the mean for age) in one of the major isotypes (IgM, IgG, and IgA) [11]. Our patient, however, only suffers from IgA deficiency and shows reasonable responses to vaccines.

The girl, therefore, only partly fits ALPS as well as CVID definitions (Table 3) and the diagnosis remains obscure. Even extensive genetic analysis did not help in this case. The ALPS-genes *FAS*, *FASLG* and *CASP10* revealed no abnormalities; the CVID-associated *TACI*-gene showed one homozygous polymorphism (Pro251Leu). This homozygous polymorphism has been identified in 6 out of 912 healthy controls from Sweden, similar to what is seen in CVID or IgA-deficient patients (Personal communication U. Salzer, Centre of Chronic Immunodeficiency, University Medical Centre, Freiburg, Germany). Moreover, recent work showed that the terminal intracellular part of *TACI* including Pro251Leu is dispensable for *TACI* signaling, which also supports the view that the Pro251Leu variation is not harmful [13]. 

Now and then, pediatric hematologists and immunologists are confronted with a patient like this without a clear diagnosis where manifestations overlap between these two adjacent fields and the clinical course is different from what is usually seen. As also shown by this girl, not all cases of autoimmune thrombocytopenia in children are self-limiting, nor all persistent profound lymphadenopathies malignant. These “hematological” features can be the first manifestation of a primary immunodeficiency syndrome. Unfortunately, this girl also illustrates that despite recent successes in further classifying primary immunodeficiencies, there are still children with clinical and laboratory features which linger between several diagnostic entities. It is important to share the medical history of these children with other specialists in the field, so that combination of experience may enhance further classification of these diseases in the future. 

## Figures and Tables

**Table 1:** Investigations in peripheral blood.

General investigations	Results	Interpretation
Hemoglobin	7.5–7.9 mmol/L	Low to normal
Neutrophils	1.3–5.3 × 10^9^/L	Intermittently low
Lymphocytes	1.6–3.2 × 10^9^/L	Low
ACE	73 U/mL	Mildly elevated
ANCA	Negative	Normal
ANA	Negative	Normal
TPO-antibodies	Negative	Normal
Thyreoglobulin antibodies	Negative	Normal
Serology rubella	IgG pos, IgM neg	Normal after vaccination
Serology CMV	IgG and IgM neg	No exposure
Serology EBV	IgG and IgM neg	No exposure
Serology HIV	Negative	No exposure
Serology parvovirus	IgG and IgM neg	No exposure
Serology bartonella henselae	IgM negative	No recent exposure
Serology toxoplasma gondii	IgG and IgM neg	No exposure
PCR blood CMV	Negative	No recent exposure
PCR blood EBV	Negative	No recent exposure
Antibody response to diphtheria and tetanus toxoid antigens	>4-fold increase in titer	Normal
Antibody response to pneumococcal polysaccharides	2-fold increase in titer	Weak response
Mantoux	Negative	Normal
M-proteins	Negative	Normal
IgM (age 7 years)	1.03 g/L	Normal
IgA (age 7 years)	0.19 g/L	Low
IgG (age 7 years)	13.4 g/L	Mildly elevated
IgG1 (age 7 years)	11.6 g/L	Mildly elevated
IgG2 (age 7 years)	0.92 g/L	Normal
IgG3 (age 7 years)	1.55 g/L	Mildly elevated
IgG4 (age 7 years)	0.058	Normal

Blood lymphocyte subpopulations	Absolute count*	Age-matched reference**

Leucocytes	4.6	9.3 (4.5–14)

Lymphocytes	1.94	2.4 (1.2–4.7)

T-lymphocytes (T) (CD3^+^)	1.01	1.80 (0.77–4.0)
Double negative TCR*αβ* ^+^ T (CD3^+^TCR*αβ* ^+^CD4^−^CD8^−^)	0.08	0.03 (0.01–0.1)
Helper-T-lymphocytes (Th) (CD3^+^CD4^+^)	0.58	0.91 (0.36–2.80)
Th naive (CD3^+^CD4^+^CD45RA^+^CD27^+^)	**0.11**	0.70 (0.20–2.50)
Th terminally differentiated (CD3^+^CD4^+^CD45RA^+^CD27^−^)	0	0.00 (0.00–0.03)
Th central memory (CD3^+^CD4^+^CD45RA^−^CD27^+^)	0.45	0.18 (0.00–0.51)
Th effector memory (CD3^+^CD4^+^CD45RA^−^CD27^−^)	0.02	0.02 (0.00–0.17)
Cytotoxic T-lymphocytes (Tc) (CD3^+^CD8^+^)	**0.19**	0.60 (0.20–1.70)
Tc naive (CD8^+^CD45RA^+^CD197^+^CD27^+^)	0.09	0.24 (0.04–1.30)
Tc terminally differentiated (CD8^+^CD45RA^+^CD197^−^CD27^−^)	**0**	0.14 (0.06–0.34)
Tc central memory (CD8^+^CD45RA^−^CD197^+^CD27^+^)	0.08	0.02 (0.01–0.04)
Tc effector memory (CD8^+^CD45RA^−^CD197^−^CD27^−^)	0.2	0.14 (0.05–0.41)
Recent thymic emigrants (CD3^+^CD4^+^CD45RA^+^CD31^+^)	**0.1**	0.59 (0.20–1.70)
Regulatory T (CD3^+^CD4^+^CD25^++^CD127^−^)	**0**	0.07 (0.02–0.27)

B-lymphocytes (B) (CD19^+^)	0.45	0.29 (0.10–0.80)
Naive B (CD19^+^CD27^−^IgM^+^IgD^+^)	0.37	0.21 (0.07–0.63)
Natural effector B (CD19^+^CD27^+^IgM^+^IgD^+^)	0.06	0.03 (0.01–0.09)
Switched memory B (CD19^+^CD27^+^IgM^−^IgD^−^)	0.01	0.02 (0.01–0.05)
Transitional B (CD19^+^CD38^++^IgM^++^)	0.01	0.03 (0.01–0.07)
CD5^+^ B (CD19^+^CD5^+^)	0.11	0.09 (0.02–0.46)
CD10^+^ B (CD19^+^CD10^+^)	0.08	0.05 (0.01–0.21)

NK-cells (CD3^−^/CD16 and/or 56^+^ cells)	0.14	0.20 (0.07–0.59)

	Ratio	Age-matched reference**

TCR-*αβ*/TCR-*γδ*	**32.7**	9.4
Th/Tc	**3.1**	1.7
*κ*/*λ*	1.14	1.5

	Percentage	Reference

TACI^+^ cells	10.6	2.6 (1–12.6)∗∗∗
BAFF-R^+^ cells	99.5	>95∗∗∗∗

All investigations were performed at the age of 5 years, unless otherwise stated. ACE: angiotensin converting enzyme; ANCA: antineutrophil cytoplasmic antibody; ANA: antinuclear antibody; BAFF: B-cell activating factor; CMV: cytomegalovirus; EBV: epstein-barr virus; HIV: human immunodeficiency virus; Ig: immunoglobulin; neg: negative; NK: Natural Killer; R: receptor; pos: positive; SD: standard deviation; TACI: transmembrane activator and calcium-modulator and cyclophilin ligand interactor; Tc: T-cytotoxic; TCR: T-cell receptor; Th: T-helper; TPO-antibodies: thyroid peroxidase antibody. ∗×10^9^/L, values in bold represent values that fall outside of the normal range. ∗∗Mean, 90% range. ∗∗∗Mean; total range. ∗∗∗∗Total range. For T-lymphocyte reference values see [3], for B-lymphocyte reference values see [2].

**Table 2:** Used antibodies.

Antibody	Source∗
Determination of blood lymphocyte subpopulations	

CD3, CD4, CD5, CD8, CD10, CD14, CD16, CD19, CD20, CD21, CD24,	Becton Dickinson
CD25, CD27, CD31, CD38, CD45, CD45RA, CD45RO, CD56, CD127,	
CD197, CD268, TCR*αβ*, TCR*γδ*, cylgG1	
CD185	Research and Diagnostics Systems
cyCD257	eBioscience
CD267 (biotin)	PeproTech
IgD, IgM, Kappa, Lambda	Dakopatts

Immunohistochemical investigations	

CD2 (AB75, 1:200), CD4 (1H6), CD8 (C8/144B, 1:50), CD23 (1B12),	Monosan
CD56 (123C3.D5), BCL2 (124)	
CD3 (polyclonal), CD5 (4C7), CD10 (56C6), CD15 (MMA), CD21 (2G9),	Ventana
CD30 (BER-H2),	
CD45 (RP2/18), CD79a (JCB117), ALK (ALK-01), Mib-1 (30-9)	
CD20 (L26,1:400)	Dakopatts

∗Becton Dickinson Biosciences (California, CA, USA), Research and Diagnostics Systems (Minneapolis, MN, USA), eBioscience (San Diego, CA, USA), PeproTech (Rocky Hill, CT, USA), Dakopatts (Glostrup, Denmark), Monosan (Uden, The Netherlands), and Ventana (Tucson, AZ, USA).

**Table 3:** Signs of ALPS and CVID definitions in our patient.

ALPS^10^	
Required criteria:	
Chronic nonmalignant noninfectious lymphoproliferation	+
Elevated CD3^+^TCR*αβ* ^+^CD4^−^CD8^−^ double-negative T-cells	±
Accessory criteria:	
Primary:	
Pathogenic mutation in *FAS, FASLG*, or *CASP10* genes	−
Defective lymphocyte apoptosis (in 2 separate assays)	−
Secondary:	
Elevated soluble FASL or serum interleukin-10 or interleukin-18 or serum plasma vit B12 levels	#
Typical immunohistological findings	−
Autoimmune cytopenias and elevated IgG levels	+
Family history of nonmalignant noninfectious lymphoproliferation	−

CVID	

Hypogammaglobulinemia	±(IgA deficiency)
Specific antibody deficiency	±(antipolysaccharide response decreased)
Autoantibodies	−
Malignancy	−
Lymphadenopathy	+
Splenomegaly	+

+ Present in our patient; ± partly or not consistently present in our patient; − not present in our patient; # not tested. ALPS: Autoimmune lymphoproliferative syndrome; CVID: common variable immunodeficiency disorders.

## References

[1] Blanchette V, Bolton-Maggs P (2010). Childhood immune thrombocytopenic purpura: diagnosis and management. *Hematology/Oncology Clinics of North America*.

[2] Schatorjé EJH, Gemen EFA, Driessen GA (2011). Age matched reference values for B-lymphocyte subpopulations and CVID classifications in children. *Scandinavian Journal of Immunology*.

[3] Schatorjé EJH, Gemen EFA, Driessen GA (2012). Paediatric reference values for peripheral T cell compartment. *Scandinavian Journal of Immunology*.

[4] Michel M, Chanet V, Dechartres A (2009). The spectrum of Evans syndrome in adults: new insight into the disease based on the analysis of 68 cases. *Blood*.

[5] Teachey DT (2012). New advances in the diagnosis and treatment of autoimmune lymphoproliferative syndrome. *Current Opinion in Pediatrics*.

[6] Seif AE, Manno CS, Sheen C, Grupp SA, Teachey DT (2010). Identifying autoimmune lymphoproliferative syndrome in children with Evans syndrome: a multi-institutional study. *Blood*.

[7] Ramyar A, Aghamohammadi A, Moazzami K (2008). Presence of idiopathic thrombocytopenic purpura and autoimmune hemolytic anemia in the patients with common variable immunodeficiency. *Iranian Journal of Allergy, Asthma and Immunology*.

[8] Teachey DT, Manno CS, Axsom KM (2005). Unmasking Evans syndrome: T-cell phenotype and apoptotic response reveal autoimmune lymphoproliferative syndrome (ALPS). *Blood*.

[9] Bussone G, Mouthon L (2009). Autoimmune manifestations in primary immune deficiencies. *Autoimmunity Reviews*.

[10] Oliveira JB, Bleesing JJ, Dianzani U (2010). Revised diagnostic criteria and classification for the autoimmune lymphoproliferative syndrome (ALPS): report from the 2009 NIH International Workshop. *Blood*.

[11] Mohammadi J, Liu C, Aghamohammadi A (2009). Novel mutations in TACI (TNFRSF13B) causing common variable immunodeficiency. *Journal of Clinical Immunology*.

[12] Narra MB, Abdou NI (2007). Autoimmune lymphoproliferative syndrome in a patient with common variable immunodeficiency: dichotomy of apoptosis. *Annals of Allergy, Asthma and Immunology*.

[13] He B, Santamaria R, Xu W (2010). The transmembrane activator TACI triggers immunoglobulin class switching by activating B cells through the adaptor MyD88. *Nature Immunology*.

